# The Nitro-Oxidative Response Is Induced in the Leaves of Barley Plants Exposed to Barium

**DOI:** 10.3390/ijms26104661

**Published:** 2025-05-13

**Authors:** Justyna Fidler, Marta Gietler, Jakub Graska, Małgorzata Nykiel, Julia Michalska, Julia Niziuk, Emilia Pełszyk, Zuzanna Ewa Perkowska, Mateusz Labudda

**Affiliations:** Department of Biochemistry and Microbiology, Institute of Biology, Warsaw University of Life Sciences-SGGW, 02-776 Warsaw, Poland; justyna_fidler@sggw.edu.pl (J.F.); marta_gietler@sggw.edu.pl (M.G.); jakub_graska@sggw.edu.pl (J.G.); malgorzata_nykiel@sggw.edu.pl (M.N.); michalskaj122@gmail.com (J.M.); j.niziuk03@gmail.com (J.N.); emka3939@gmail.com (E.P.);

**Keywords:** barium, nitric oxide, reactive oxygen species, nitro-oxidative stress, barley

## Abstract

Barium (Ba) is classified as a non-essential element, meaning that it does not play a requisite role in the physiological processes of living organisms, but it poses a significant health risk to them. Plants that grow in Ba-rich soils, particularly near barite outcrops or mining waste, often accumulate high levels of Ba. Excess Ba in plant cells can lead to the overproduction of reactive oxygen species (ROS), which contributes to oxidative stress. Typically, nitric oxide (NO) can help alleviate heavy metal stress; however, under certain conditions, elevated levels of superoxide and nitric oxide may result in nitrosative and nitrative stress. This study investigated whether exposing barley plants to barium acetate (300 μM and 600 μM) triggers a nitro-oxidative response in spring barley plants. The molecular and biochemical analyses revealed fluctuations in the gene expression and activity of antioxidant enzymes and a steady rise in hydrogen peroxide (H_2_O_2_) in the leaves. Lower Ba concentrations and shorter exposures increased NO levels, while higher concentrations and more prolonged exposure reduced them, affecting nitrogen metabolism. These findings highlight the toxicological risks posed by Ba, especially for cultivated plants, and underscore the need for further research on its impact on plant physiology and the potential risks to human health.

## 1. Introduction

Barium (Ba) is a dense alkaline earth metal that shares similarities with calcium and magnesium [1,2]. Despite its metallic properties, Ba is deemed non-essential for living organisms and presents significant health risks [3]. Barium can pose a challenge to plants and animals. Although the impact of Ba on human health has been partially studied, its effects on plants are much less well-known.

Exposure to Ba can result in a range of harmful effects on humans, particularly impacting the cardiovascular and renal systems. Health issues linked to Ba exposure may include cardiac arrhythmia, acute hypertension, and kidney dysfunction, such as hypokalemia, as well as gastrointestinal disturbances. In severe cases, Ba exposure can lead to neurological disorders and, ultimately, may even be fatal. Research indicates that Ba has the potential to induce oncogenic changes in human cells and to amplify the carcinogenic effects of arsenic [4,5,6,7,8]. Additionally, Ba exposure can disrupt the delicate equilibrium between pro-apoptotic and anti-apoptotic signaling pathways, thereby hindering the apoptosis of cancerous cells [9,10].

In addition to being a health hazard, Ba also has a negative effect on plant growth and development. The risk of plant exposure to Ba is increased by industrial activities, primarily the mining and refining of Ba ore and the production of Ba salts [2]. Over the past fifty years, Ba compounds have been extensively utilized across various industrial and medical sectors, such as the petroleum and steel industries, as well as gastrointestinal radiography [7]. Furthermore, Ba is utilized in the production of industrial soaps, explosives, fire extinguishers, drilling fluids, and insecticides; therefore, the area potentially exposed to Ba contamination is quite large.

Plants growing in soils with high Ba content, particularly in areas near barite (BaSO_4_) outcrops or mine spoils, can themselves exhibit elevated levels of Ba [1]. Studies have shown that the concentration of Ba in the aboveground tissues of these plants can match or even surpass that found in their root systems [11]. Interestingly, some plant species, like *Indigofera cordifolia*, have demonstrated remarkable resilience to elevated levels of trace metal elements, enabling them to thrive in environments adversely affected by barite, although they accumulated high Ba contents [12]. Moreover, the increasing intensity of atmospheric phenomena linked to climate change, such as powerful winds and heavy rainfall, could transport Ba particles into neighboring agricultural areas, thereby being a risk factor for crops. This concern remains insufficiently examined, and there is a significant gap in the research in regard to addressing the effects of Ba exposure on crops, especially in those crops that are important from the point of view of food and feed security. Barley (*Hordeum vulgare* L.), one of the first domesticated crops, ranks fourth in the world in terms of crop production, after wheat, rice, and maize. It is cultivated worldwide in many countries and regions, mainly in temperate climates in both the northern and southern hemispheres [13]. Europe is estimated to account for about 65% of world barley production. Barley was originally used as a food for humans and, later, with the increasing use of wheat in the human diet, barley came to be used as animal feed, as well as a key crop for malting [14]. It is noteworthy that because barley is a diploid, it is also an important model plant for research on the molecular aspects, biochemistry, and developmental biology of cereals [15].

Exposure to Ba and its accumulation in plant cells beyond a specific threshold can lead to the overproduction of reactive oxygen species (ROS). Similarly, under normal cell conditions, nitric oxide (NO) concentrations are typically in the nanomolar range, serving as a sink for superoxide and mitigating oxidative stress. However, in certain pathological conditions, both superoxide and NO concentration levels can rise, resulting in nitrosative and nitrative stress, which often precedes oxidative stress. When superoxide levels equal or exceed those of NO, oxidative stress occurs. Hence, the co-occurrence of nitrosative and oxidative stress, and their undoubted interaction prompted some scientists to formulate the new term: “nitro-oxidative” stress [16]. Based on our previous findings indicating significant redox disturbances in plants subjected to various environmental stresses, we formulated a research hypothesis that the exposure of barley plants to elevated Ba concentrations triggers a nitro-oxidative response. We want to stress the importance of this study to researchers of plant stress biology, particularly those focused on redox mechanisms. The proposed integrated approach could uncover previously unknown mechanisms that drive responses to redox imbalances caused by various environmental challenges, significantly advancing our understanding of these complex processes.

## 2. Results and Discussion

**Figure 1:**
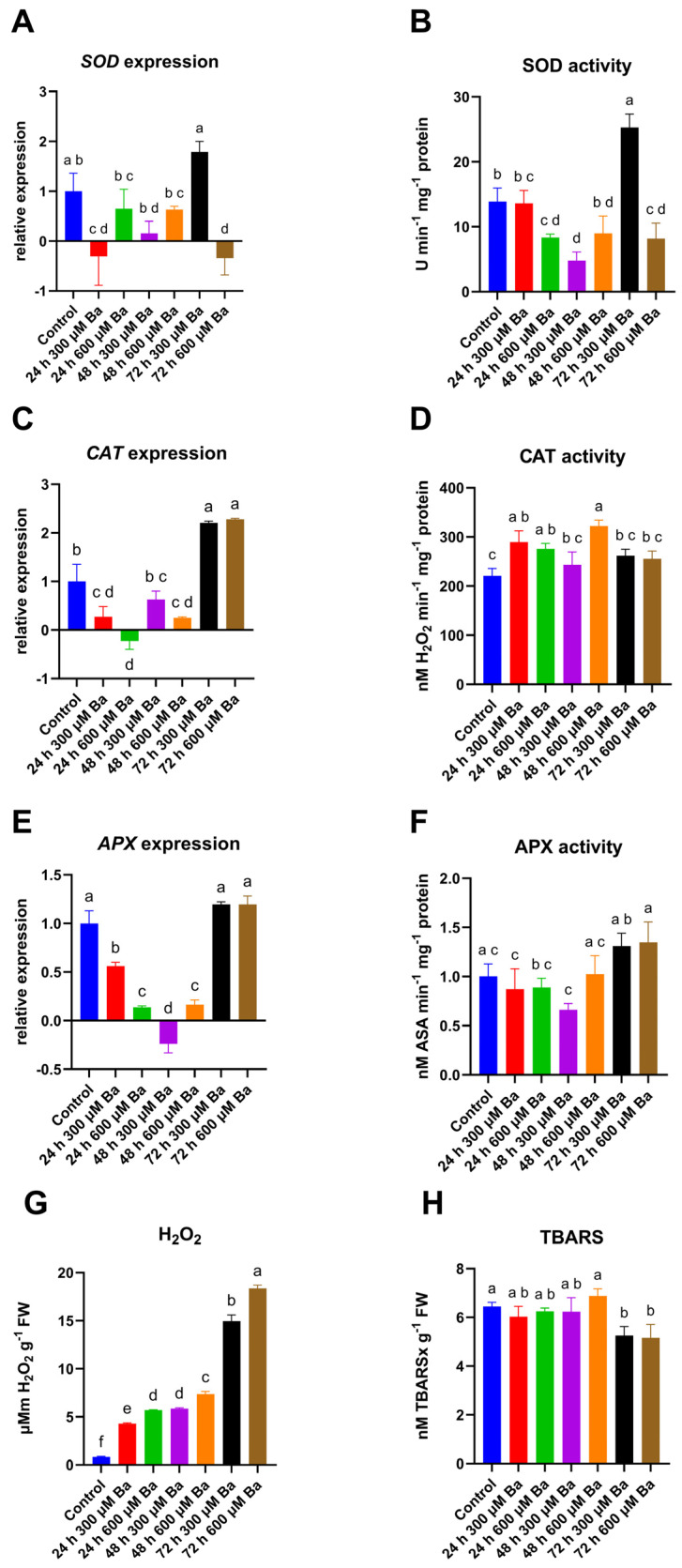
The expression levels of superoxide dismutase (*SOD*) (**A**), the activity of SOD (**B**), the expression levels of catalase (*CAT*) (**C**), the activity of CAT (**D**), the expression levels of ascorbate peroxidase (*APX*) (**E**), the activity of APX (**F**), the hydrogen peroxide (H_2_O_2_) content (**G**), and the amount of 2-thiobarbituric acid reactive substances (TBARS) (**H**), in the leaves of barley plants treated with the Ba solution. The results are shown as the mean  ±  SD. Different letters indicate homogeneous groups that are significantly different at *p* < 0.05, according to a two-way analysis of variance and a post hoc Tukey’s test.

The expression of genes that encode antioxidant enzymes, namely *SOD*, *CAT*, and *APX*, was changed by the Ba treatment and was lower than in the control for the first 48 h of treatment. *SOD* expression (Figure 1A) in the 300 µM Ba treatment initially decreased abruptly after 24 h of treatment and then increased gradually to reach a level higher than the control after 72 h, although this finding was not statistically significant. The opposite trends were observed in the 600 µM Ba treatment, where a greater decrease in *SOD* expression was observed with time, reaching the lowest value after 72 h of treatment. The changes in SOD activity (Figure 1B) showed similar trends. Although the decrease in SOD activity with the 300 µM Ba treatment was noticeable only after 48 h, it was probably due to slow enzyme degradation, while its synthesis was inhibited [17]. However, at 72 h, the SOD activity increased by more than 80% compared to the control, which corresponded to its high expression at this time point. Higher Ba concentrations caused a faster decrease in SOD activity, which was already visible after 24 h. The lower SOD activity due to the 600 µM Ba treatment was maintained throughout the experiment, reaching a minimum level at 72 h, which was about 40% lower than the control.

*CAT* expression (Figure 1C) was lower than in the control for both treatments for the first 48 h, then increased significantly after 72 h of treatment, reaching a value twice as high as the control. However, the CAT activity did not fluctuate as much (Figure 1D). For the 300 µM Ba treatment, the CAT activity was the highest after 24 h, then it decreased, but it remained 11–18% higher than in the control, although these differences were not statistically significant. For the 600 µM Ba treatment, a steady increase in CAT activity was observed for 48 h (after 24 h by 25%, after 48 h by 46%), while after 72 h, despite the increase in expression, there was a decrease in activity, which remained slightly higher than the control, although the difference was not significant.

The *APX* expression (Figure 1E) in the treatment with 300 µM Ba gradually decreased for the first 48 h, reaching the lowest value at 48 h. In the treatment with 600 µM Ba, a more rapid decrease in relative expression was observed, which remained at a constant level for 48 h. On the third day, the level of relative *APX* expression in both treatments returned to a level similar to the control. The changes in APX activity showed similar trends to the expression changes, but they were much milder (Figure 1F). At 24 h, at both Ba concentrations, the APX activity was reduced by about 12%. At 48 h, the APX activity at a concentration of 300 µM Ba decreased by 34%, while at 600 µM Ba it increased to the control values. After 72 h, the APX activity at both Ba concentrations was comparable and about 31–34% higher than in the control; however, the change was not significant.

The literature on the effect of Ba on redox homeostasis in plants is very limited. The results obtained so far mainly concern the study of antioxidant enzyme activity. It was observed that the activity of antioxidant enzymes in cucumber and soybean plants changed under the influence of Ba. The CAT activity increased in old leaves and the stems of cucumber plants [18]. Similar results were obtained in soybean plants, where the CAT activity increased under the influence of long-term Ba stress [19]. In our experiment, we observed an increase in CAT activity; however, the high expression combined with relatively low activity of CAT at 72 h may be linked to the structural misfolding of the enzyme due to its interaction with Ba. This phenomenon is similar to what has been observed with cadmium (Cd) [20]. In turn, in regard to Ba treatment, the APX activity increased in cucumber stems and roots, but a decrease in the activity of this enzyme was observed in young and old leaves [18]. It seems that in barley, the APX activity after treatment with heavy metals depends on the metal concentration, as well as on the resistance of the cultivar and the plant’s growth phase. In the Tombari cultivar, the APX activity remained at the same level at both cadmium concentrations of 15 and 30 mg/kg [21]. Similarly, in the study on four cultivars of spring barley (Simfoniya, Mestnyj, Ca 220702, Malva) in most of them no significant differences in the APX activity in response to Cd were demonstrated [22].

An increase in the total SOD activity was observed in soybean plants in response to Ba exposure in the soil [19]. The dependence of SOD activity on the concentration of heavy metals was also demonstrated in barley treated with Cd. Lower doses caused no changes or a slight increase in the activity of this enzyme, while at high doses the inhibition of SOD activity was observed [23]. Similarly, in our studies, longer exposure to higher concentrations of Ba resulted in a decrease in SOD activity, while at lower concentrations, after 72 h, there was a significant increase in SOD activity.

It has been shown that ROS are generated in response to transition heavy metals via direct electron transfer; however, Ba is not one of them. Heavy metals can also disrupt redox homeostasis, which is caused by their interaction with functional groups of membrane proteins, as well as by substituting central metals in enzymes, which can lead to electron leakage. Furthermore, heavy metals can cause the inactivation of some antioxidant enzymes or the lowering of the concentration of low-molecular-weight antioxidants (e.g., glutathione, used for the synthesis of phytochelatins) [24].

The content of H_2_O_2_, which is a marker of ROS content, increased as a result of Ba treatment (Figure 1G). The content of H_2_O_2_ increased over time and, at each measurement point, was higher in the treatment with 600 µM Ba than in the 300 µM Ba treatment. Compared to the control, after 72 h, the H_2_O_2_ content increased more than 18 times in regard to the lower dose of Ba and more than 22 times in regard to the higher dose. Different results were observed for lipid peroxidation, indicating damage to biological membranes (Figure 1H). The concentration of 2-thiobarbituric acid reactive substances (TBARS) remained at a level similar to the control for the first 48 h of treatment and then decreased by approximately 18–19% in 72 h. No differences were observed between the treatments.

Previous studies have shown an increase in H2O2 content under the influence of Ba treatment in *Panicum maximum* [25], as well as under the influence of other metals, e.g., Cd in grasses [26,27]. However, increased ROS content does not always generate oxidative damage, such as lipid peroxidation, which was observed not only in barley, but also in soybean leaves, under the influence of Ba [19]. It appears that the applied concentrations of Ba induced a response associated with antioxidant mechanisms, such as an increase in the expression of genes encoding CAT and APX, which enabled the plants to effectively protect their cellular structures from excess ROS concentrations.

Nitrogen uptake and assimilation are key processes influencing plant metabolism, thus controlling plant growth and development, as well as the adaptation to stress conditions. It has been shown that both N uptake and assimilation can be impaired by a high concentration of heavy metals in the soil [28]. Nitrite and ammonium ions are the main sources of nitrogen for plants, as they are essential for various metabolic pathways. The conversion of NO_3_^−^ to NH_4_^+^ is a two-step process. In the first step, NO_3_^−^ is converted to NO_2_^−^ by cytosolic nitrate reductase (NR) and, in the next step, NO_2_^−^ is converted to NH_4_^+^ by plastidial nitrite reductase (NiR) [29].

**Figure 2:**
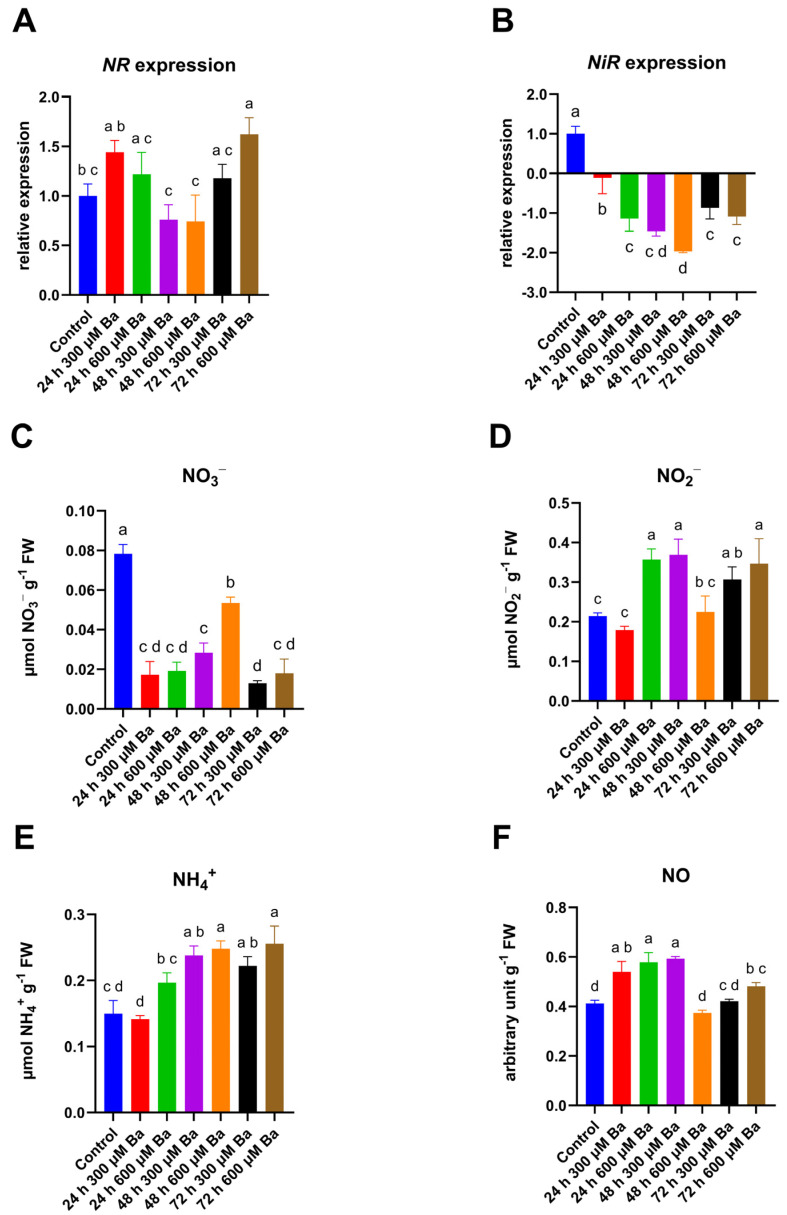
The expression levels of nitrate reductase (*NR*) (**A**) and nitrite reductase (*NiR*) (**B**) and the content of nitrate (NO_3_^−^) (**C**), nitrite (NO_2_^−^) (**D**), ammonium (NH_4_^+^) (**E**), and nitric oxide (NO) (**F**) in the leaves of barley plants treated with a Ba solution. The results are shown as the mean  ±  SD. Different letters indicate homogeneous groups that are significantly different at *p* < 0.05, according to a two-way analysis of variance and a post hoc Tukey’s test.

The relative expression of *NR* fluctuated for 48 h, but the changes were not statistically significant (Figure 2A). At 24 h, an upward trend could be observed, followed by a decrease at 48 h, and then an increase in relative expression was observed again at 72 h. In the 300 µM Ba treatment, the expression level was comparable to the control after 72 h and, in the 600 µM Ba treatment, it was about 50% higher than the control, and the increase was statistically significant. The changes in *NiR* expression were far greater (Figure 2B). The relative expression of *NiR* reached negative values for all the treatments, reaching a minimum level after 48 h in the 600 µM Ba treatment. The expression of *NiR* was lower at the same time points in the treatment containing a higher concentration of Ba.

The analyses conducted so far have shown that heavy metals cause a decrease in NR activity in various plant species. In studies conducted on *Vigna trilobata*, wherein the effect of Ba on NR activity was examined, a decrease was also observed, although it was not very significant [30]. The mechanism by which enzymatic activity is inhibited can vary. For example, under Al toxicity, the inhibition occurs due to Al’s direct interaction with the functional -SH groups in the active sites of NR. In the presence of Cd or Cr, the inhibition of NR results from changes in chlorophyll biosynthesis, photosynthesis, and sugar metabolism [31,32,33]. It should be noted that the relatively small differences in the expression of the gene encoding NR observed in our work do not necessarily directly translate into changes in the activity of this enzyme. Moreover, the activity of NR may also be inhibited by the abovementioned mechanisms. Studies on the effect of heavy metals on NiR activity are very limited, since NiR has less impact on N assimilation. NiR is located in chloroplasts; therefore, heavy metals have less access to NiR than to cytosolic NR [28]. A decrease in NiR activity is observed in connection with the occurrence of heavy metals, e.g., in wheat seedlings under the influence of As [34]. It was indicated that the reduced NiR activity is the result of the reduced expression of genes encoding this enzyme rather than modifications in protein activity.

In response to Ba, the content of individual nitrogen forms also changed. The content of NO_3_^−^ ions decreased as a result of Ba treatment (Figure 2C). After 24 h, the decrease was similar for both BA concentrations and reached about 75% of the level in the control. In the treatment with 300 µM of Ba, a reduced level was maintained throughout the experiment, while in the treatment with a concentration of 600 µM Ba, after 48 h, an increase in the content of nitrate ions was observed that reached almost 70% of the control level, which decreased again in 72 h. Different trends were noted for NO_2_^−^ ions (Figure 2D). As a result of the treatment with 300 µM Ba, the content of NO_2_^−^ ions increased significantly after 48 h and remained at a similar level for 72 h. In the treatment with 600 µM of Ba, an increase in the content of NO_2_^−^ ions was observed after 24 h, after which the content of these ions decreased to a level similar to the control and increased again for 72 h. The content of NH_4_^+^ ions also changed over time in response to the treatment with Ba (Figure 2E). For the treatment with a concentration of 300 µM Ba, changes analogous to those in regard to the level of NO_2_^−^ ions were observed, i.e., an increase in 48 h, which was maintained until the end of the experiment. As a result of the exposure to the treatment with 600 µM of Ba, increasing trends were observed after 24 h, but they were not statistically significant. A clear increase was observed after 48 h, and it was maintained until the end of the experiment. There were no differences in the NH_4_^+^ levels between the different Ba concentrations at 48 h and 72 h. There were also changes in the NO content (Figure 2F), which were dependent on the Ba concentration and duration of the treatment. For the treatment with a concentration of 300 µM Ba, an increase in the NO content of over 30% was observed, which was maintained for 48 h, after which it decreased to the control level. As a result of the application of the 600 µM Ba treatment, an increase in NO content by 40% was observed after 24 h, a decrease after 48 h to the control level, and a further, but smaller, increase by 17% was recorded compared to the control after 72 h of treatment.

The NO_3_^−^ content varies under the influence of heavy metals. In rice treated with Ni, similar effects to those observed in our experiment with Ba were noted, where the NO_3_^−^ content decreased and the NH_4_^+^ content increased [35]. Similar changes were also observed in other plant species [36], for example in *Triticum aestivum* [37], when exposed to Ni, as well as in *Phaseolus vulgaris* treated with Cd [38]. The decrease in NO_3_^−^ content may stem from the reduced uptake and transport of NO_3_^−^ from the roots. Conversely, in *Vigna trilobata* and *Cyamopsis tetragonoloba* exposed to high concentrations of Ba, an increase in NO_3_^−^ content was observed [30,39]. However, there is currently no data on the effect of Ba on other forms of nitrogen content. Increased NH_4_^+^ accumulation can potentially be toxic and may lead to an osmotic imbalance and, consequently, metabolic disruptions [36]. The effect of Ba on NO_2_^−^ content has yet to be described. It has been demonstrated that Cd stress significantly reduces the NO_2_^−^ content in rice, while Mo has the opposite effect. NO_2_^−^ is a molecule that can be converted to NH_4_^+^ by NiR, but can also be transformed into NO by NR [40]. Therefore, the balance between NO_3_^−^, NO_2_^−^, NH_4_^+^, and NO is complex and multifaceted. Nitric oxide plays a role in several plant developmental processes (e.g., root hair development), as well as in phytohormone and ROS signaling and helps to mitigate the effects of stress, including heavy metal toxicity. In response to heavy metals, NO reduces ROS levels by enhancing the activity of antioxidant enzymes and forming less stable peroxynitrite [41]. It has been found that Cd, As, Cu, Pb, Al, Zn, and Ni influence NO production; however, whether they increase or decrease NO content depends on the specific plant, organ, heavy metal concentration, and the duration of the treatment [42]. To date, the effect of Ba on NO accumulation has not been documented in any plant. Our studies suggest that lower Ba concentrations and shorter exposure times favor NO accumulation in barley, while higher concentrations and prolonged treatment result in decreased levels of this molecule, reducing it to values close to the control level or slightly above it.

**Figure 3:**
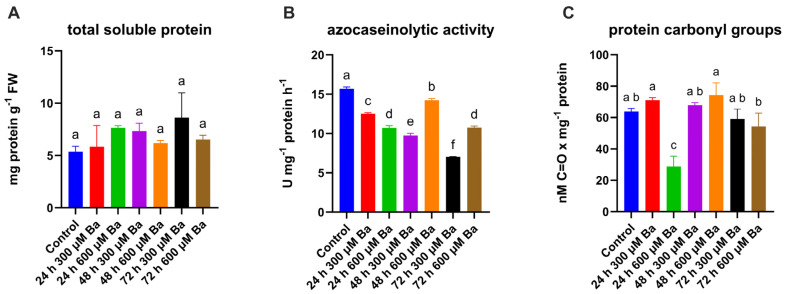
The contents of total soluble protein (**A**), azocaseinolytic activity (**B**), and content of protein carbonyl groups (**C**) in the leaves of barley plants treated with a Ba solution. The results are shown as the mean  ±  SD. Different letters indicate homogeneous groups that are significantly different at *p* < 0.05, according to a two-way analysis of variance and a post hoc Tukey’s test.

The content of soluble protein increased slightly as a result of stress, but these changes were not statistically significant (Figure 3A). The highest protein content was observed after the application of the 300 µM Ba treatment for 72 h and was higher by about 60% than the control.

A significant change was observed in the azocaseinolytic activity (Figure 3B), which, in contrast to the soluble protein content, decreased in response to the Ba treatment. In regard to the 300 µM Ba treatment, the azocaseinolytic activity decreased steadily and, after 24 h, was lower by 19% than in the control, after 48 h by 38%, and after 72 h by 55%. The highest protein concentrations were observed with the lowest proteolytic activity. At a higher concentration of Ba, the initial decrease was stronger and amounted to over 31%; however, after 48 h, it increased, reaching a value close to the control, then decreased again and was almost identical to the value after 24 h of treatment. The content of carbonylated proteins, which are commonly considered to be a marker of oxidative damage to proteins, also changed (Figure 3C). At a concentration of 300 µM Ba, the content of carbonylated proteins increased slightly for the first 24 h, then decreased; however, the observed differences were not statistically significant compared to the control. Greater fluctuations were observed in the treatment with a concentration of 600 µM Ba, wherein, after 24 h, the content of carbonylated proteins was 57% lower than in the control, then it increased rapidly, reaching the maximum level after 48 h of treatment; after 72 h, the content of oxidized proteins was comparable in both treatments.

The content of soluble protein can indicate reversible and irreversible metabolic changes in response to heavy metal stress [43], as well as serve as one of the key osmoprotectants [44]. Research shows that maize varieties more tolerant to heavy metals (Cd, As) exhibit greater accumulation of soluble proteins, while sensitive varieties do not show such accumulation [44]. A study on the effect of Ba on *Cyamopsis tetragonoloba*, *Vigna trilobata*, *Pigeon Pea*, and *Triticum aestivum* revealed a decrease in protein content with an increasing Ba concentration; however, the applied Ba concentrations were much higher than those used in the other studies presented [30,39,45,46]. This may indicate similar trends to those observed in studies on *Aspergillus niger*, wherein low concentrations of heavy metals led to an increase in the soluble protein content, while high concentrations resulted in a decrease [47]. Despite the lack of significant changes in the protein content, notable differences in regard to proteolysis were observed, which was reduced. There is currently no information on the effect of Ba on proteolysis and carbonylation in plants; however, it has been shown that other heavy metals can influence these processes. In rye and triticale, Al treatment increased proteolysis [48]. The impact of heavy metals on proteolysis, however, is dependent on the type of metal. In studies on sunflower plants, among the metals tested, 100 μM of AlCl_3_, CoCl_2,_ CuCl_2_, CrCl_3_, HgCl_2_, NiCl_2_, PbCl_2_, and ZnCl_2_, only Zn was found to increase proteolysis. At the same time, in these plants, only Co^2+^, Cu^2+^, Hg^2+^, and Ni^2+^ were observed to increase the content of carbonyl groups in proteins [49]. On the other hand, studies on *Alyssum montanum* shoots growing in the presence of heavy metals demonstrated an increased content of carbonylated proteins [50]. In our studies, we showed that Ba at both concentrations of 300 µM and 600 µM reduced protein hydrolysis, which intensified with the duration of stress, but it did not increase the oxidative damage to proteins.

**Figure 4:**
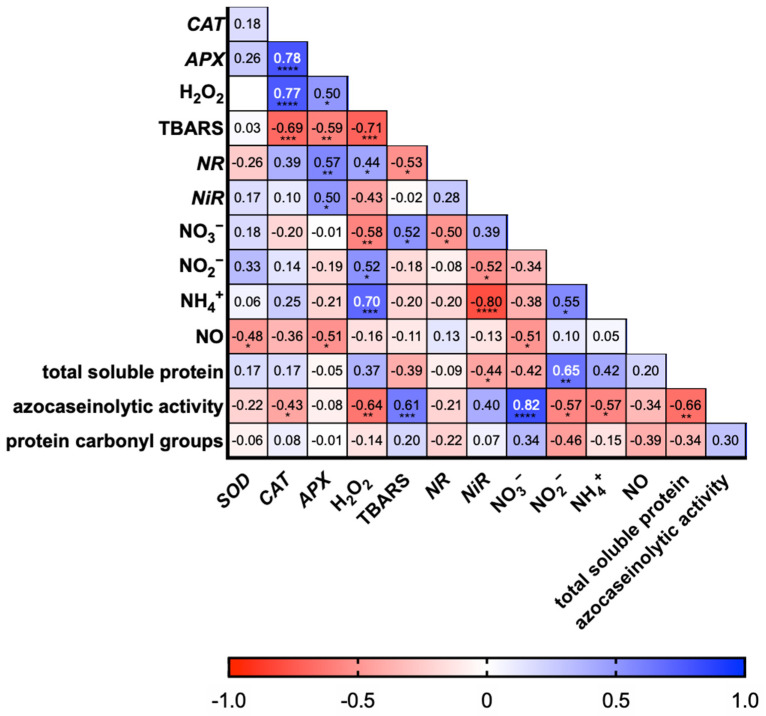
A heat map on the correlation coefficients between *SOD*, *CAT*, *APX*, H_2_O_2_, TBARS, *NR*, *NiR*, NO_3_^−^, NO_2_^−^, NH_4_^+^, NO, total soluble protein, azocaseinolytic activity, and protein carbonyl groups. Blank space means a value of correlation close to zero; ****—a significance level of *p* ≤ 0.0001, ***—a significance level of *p* ≤ 0.001, **—a significance level of *p* ≤ 0.01, and *—a significance level of *p* ≤ 0.05.

The analysis of the correlation coefficients showed connections between the parameters studied (Figure 4). The highest positive correlation was found between the NO_3_^−^ ion content and azocaseinolytic activity (0.82), *APX* and *CAT* expression (0.78), *CAT* expression and H_2_O_2_ content (0.77), and NH_4_^+^ and H_2_O_2_ ion content (0.70). The strongest negative correlations were observed for *NiR* expression and NH_4_^+^ content (−0.80), and TBARS and H_2_O_2_ content (−0.71). Lower, but significant, positive correlation coefficient values were also noted for: total soluble protein and NO_2_^−^ (0.65), azocaseinolytic activity and TBARS (0.61), NR and APX expression (0.57), NH_4_^+^ and NO_2_^−^ ion content (0.55), NO_2_^−^ and H_2_O_2_ content (0.52), NO_3_^−^ and TBARS content (0.52), H_2_O_2_ content and *APX* expression (0.50), *NiR* and *APX* expression (0.50), and NR expression and H_2_O_2_ content (0.44). Other negative, significant correlation coefficients were found for: TBARS content and *CAT* expression (−0.69), total soluble protein and azocaseinolytic activity (−0.66), H_2_O_2_ content and azocaseinolytic activity (−0.64), TBARS content and *APX* expression, NH_4_^+^/NO_2_^−^ ion content and azocaseinolytic activity (−0.57), *APX* expression and NO content (−0.51), *NR* expression and NO_3_^−^ content, *SOD* expression and NO content (−0.48), *NiR* expression and total protein (−0.44), and *CAT* expression and azocaseinolytic activity (−0.43). The only factor that did not show significant correlations with the remaining studied parameters was protein carbonylation.

The performed correlation analysis suggests that the relationship between ROS, protein, and nitrogen metabolism is complex. At the same time, the lack of studied interactions indicates the need for further research that would explain these strict dependencies. While there are premises for the occurrence of interactions between, for example, NO and ROS [41], the remaining important dependencies do not seem to be directly related. It is not without significance that, especially in cultivated plants, Ba toxicity can have a direct effect on farm animals and humans, which is why there is a need to continue research in this field [5].

## 3. Materials and Methods

### 3.1. Plant Material

The study was performed on spring barley seedlings (*Hordeum vulgare* L.) (BBCH 13), the Tilmor variety (provided by Danko Plant Breeders Ltd., Choryń, Poland), subjected to Ba in the form of barium acetate (Ba(C_2_H_3_O_2_)_2_). The seeds were germinated between two strips of filter paper (22 cm × 8 cm), rolled into cylindrical assemblies and, subsequently, incubated for 10 days in Knop’s solution, supplemented with Hoagland microelements. These experiments were conducted within a controlled environment in a climate chamber (Versatile Environmental Test chamber, MLR-325H, Panasonic), maintaining a temperature of 23 ± 2 °C for 16 h (day) and 18 °C for 8 h (night). The photosynthetic photon flux density (PPFD) was 100 ± 25 μmol m^−2^ s^−1^, with a relative humidity maintained at 70–80% throughout the experimental period.

On the tenth day, a part of the seedlings was harvested to serve as a control sample, then frozen in liquid nitrogen, and stored at −80 °C for subsequent analysis. The remaining seedlings were divided, with one group transferred into a substrate solution containing 300 μM barium acetate, while the other was transferred into a substrate solution containing 600 μM barium acetate. After a period of 24, 48, and 72 h, a selection of these plants was harvested and preserved identically to the control samples.

### 3.2. Hydrogen Peroxide Content

The hydrogen peroxide (H_2_O_2_) levels were determined following the method proposed by Alexieva et al. [51]. Leaf tissues (100 mg) were homogenized in an ice bath, using 1 mL of 0.1% (*w*/*v*) trichloroacetic acid (TCA). The homogenate was then centrifuged at 12,000× *g* for 15 min. A total of 25 μL of the supernatant was combined with 25 μL of 10 mM potassium phosphate buffer (pH 7.0) and 50 μL of 1 M KI. The measurement was performed on a Nunc U-bottom 96-well plate (Thermo Scientific, Waltham, MA, USA), using a Varioskan LUX Multimode Microplate Reader (Thermo Scientific). The absorbance was recorded at 390 nm, and the content of H_2_O_2_ was calculated as μM × g^−1^ FW, based on a standard curve.

### 3.3. Lipid Peroxidation

Lipid peroxidation was measured according to the method described by Heath and Packer [52]. Fresh leaves (100 mg) were homogenized in 0.1% trichloroacetic acid (TCA, *w*/*v*). The resulting homogenate was then centrifuged at 4 °C for 10 min at 13,200× *g*. From the supernatant, 0.5 mL was combined with 1.5 mL of 0.5% (*w*/*v*) 2-thiobarbituric acid (TBA) dissolved in 20% TCA. This mixture was incubated at 95 °C for 25 min. After incubation, the samples were cooled on ice, and absorbance was measured at 532 nm and 600 nm (to account for nonspecific turbidity). The determination was performed on a Nunc U-bottom 96-well plate, using a Varioskan LUX Multimode Microplate Reader. The lipid peroxidation results were expressed as TBARS, calculated using an extinction coefficient of 155 mM^−1^ × cm^−1^, and reported as nM × g^−1^ fresh weight (FW).

### 3.4. Nitrogen Metabolite Content

Nitric oxide (NO) formation was quantitatively assessed utilizing DAF-FM™ diacetate (4-amino-5-methylamino-2′,7′-difluororescein diacetate; Invitrogen Molecular Probes) (a modified version of the method by Arasimowicz-Jelonek et al. [53]). Leaf tissues, weighing 100 mg, were submerged in 1 mL of a 100 mM phosphate buffer (pH 7.4), supplemented with 15 μM of DAF-FM™ diacetate. The samples were agitated at 300 rpm for 1 h in darkness at room temperature to facilitate the extraction of NO. Following incubation, the samples were subjected to centrifugation at 9000× *g* for 10 min at room temperature to remove the tissue debris. The supernatant was carefully transferred to a 96-well black plate (Thermo Scientific) for subsequent analysis. NO production was measured on black 96-well Immuno plates (Thermo Scientific), using a Varioskan LUX Multimode Microplate Reader, with excitation at 485 nm and emission at 515 nm. The results on the nitric oxide formation were expressed in arbitrary units of fluorescence intensity (U) per gram of fresh weight (U × g^−1^ FW).

The extraction process for determining the ammonium, nitrate, and nitrite contents was performed according to a modified version of the method by Huang et al. [54]. Leaf samples (100 mg) were immersed in 1 mL of deionized water and shaken at 1000 rpm for 1 h at 45 °C. After shaking, the samples were centrifuged at 16,000× *g* for 20 min at 4 °C, and the supernatants were collected.

The ammonium (NH_4_^+^) content was determined by adding 10 μL of the supernatant to 190 μL of Nessler reagent (Sigma-Aldrich, Saint Louis, MO, USA). The homogenates were incubated for 20 min at room temperature on a Nunc U-bottom 96-well plate. Absorbance was then measured at 404 nm, using a Varioskan LUX Multimode Microplate Reader. The ammonium content was calculated using ammonium acetate to create a standard curve and was expressed as μmol per gram of fresh weight (μM × g^−1^ FW).

The content of nitrates (NO_3_^−^) and nitrites (NO_2_^−^) was determined using the Nitric Oxide Assay Kit (Invitrogen/Thermo Scientific, Waltham, MA, USA), following the manufacturer’s instructions. The absorbance of both compounds was measured photometrically at 540 nm. The determination was performed on a Nunc U-bottom 96-well plate, using a Varioskan LUX Multimode Microplate Reader. To calculate the results, standard curves were prepared using the standards provided in the kit. Nitrate and nitrite concentrations were expressed as µM × g⁻^1^ FW.

### 3.5. Protein Extraction

Protein extracts were prepared according to the method described by Labudda et al. (2018) [55], with some modifications. Leaf samples (100 mg) were homogenized in 1 mL of an ice-cold extraction buffer that contained 50 mM potassium phosphate buffer (pH 7.0), 2 mM 2-mercaptoethanol, 0.1 mM EDTA, 0.5% (*w*/*v*) Triton X-100, 2% (*w*/*v*) polyvinylpyrrolidone (PVP), and 1 mM phenylmethylsulfonyl fluoride (PMSF). The homogenates were incubated on ice for 20 min and then centrifuged at 16,000× *g* for 15 min at 4 °C.

The total soluble protein content was measured using Coomassie Brilliant Blue G-250 stain, following the protocol described by Spector (1978) [56], with bovine serum albumin as the protein standard. The total soluble protein content was expressed in mg of protein per gram of fresh weight (mg × g^−1^ FW).

### 3.6. Azocaseinolytic Activity

Leaf tissue (1 g) was ground into a powder in liquid nitrogen and extracted using 5 mL of pre-cooled extraction buffer (Tris–HCl, 50 mM, pH 7.2), containing 5 mM β-mercaptoethanol and 0.2 g of insoluble PVP. The homogenate was then filtered and centrifuged at 15,000× *g* for 10 min at 4 °C. The supernatant was utilized for the enzyme assay. The reaction mixture consisted of 50 μL of the enzyme extract, 0.15 mL of 0.5% (*w*/*v*) azocasein, and 0.3 mL of citrate buffer (0.25 M, pH 5.0). After incubating for 2 h at 37 °C, the reaction was halted by adding 1 mL of 12% (*w*/*v*) TCA. The acid-soluble products were measured spectrophotometrically at 340 nm. One unit of azocaseinolytic activity was defined as the amount of enzyme that produced a 0.01 increase in absorbance. It was expressed as U × mg^−1^ protein × h^−1^.

### 3.7. Measurements of Antioxidant Enzyme Activity

The activity of SOD (EC 1.15.1.1) was measured following the protocol described by Kostyuk and Potapovich (1989) [57]. An assay buffer was prepared by mixing equal volumes of 67 mM potassium/sodium phosphate buffer (pH 7.8) and 25 mM EDTA. The pH of this solution was adjusted to 10.0 using TEMED. Next, 200 μL of the assay buffer was combined with 20 μL of a supernatant that had been previously diluted with Milli-Q water at a ratio of 1:10. The SOD enzymatic assay was initiated by adding 20 μL of 2.5 μM quercetin into DMSO. The determination was conducted on a Nunc U-bottom 96-well plate, using a Varioskan LUX Multimode Microplate Reader. The absorbance of the samples was recorded immediately and again after 20 min at 406 nm. SOD activity was expressed in arbitrary units, representing the amount of SOD that inhibits superoxide-driven oxidation of quercetin by 50% per min, per mg of protein.

The CAT (EC 1.11.1.6) activity was measured following the method by Góth (1991) [58], with some modifications. The reaction was initiated by adding 5 μL of the supernatant to 1 mL of a 0.03% (*v*/*v*) hydrogen peroxide (H_2_O_2)_ solution, in 60 mM of Tris-HCl buffer, at pH 7.0. The samples were incubated at 37 °C for 3 min. After incubation, 0.5 mL of 4% (*w*/*v*) ammonium molybdate dissolved in 0.250 M sulfuric acid (H_2_SO_4_) and 0.450 mL of 0.25 M H_2_SO_4_ were added to the samples. The samples were then centrifuged for 5 min at 16,000× *g*, and the absorbance of the supernatant was measured at 405 nm. Catalase activity was expressed as nM H_2_O_2_ × min^−1^ × mg^−1^ protein.

The APX (EC 1.11.1.11) activity was measured following the method described by Nakano and Asada (1981) [59]. Five μL of the enzymatic extract was mixed with a medium containing 50 mM Tris-HCl buffer (pH 7.2), 2 mM ascorbic acid (AsA), 5 mM EDTA, and 0.1 mM hydrogen peroxide (H_2_O_2_). The APX activity was assessed at 25 °C on a Nunc U-bottom 96-well plate, using a Varioskan LUX Multimode Microplate Reader. The absorbance was recorded at 290 nm over a period of 5 min, with readings taken every minute. APX activity was expressed in nM AsA × min^−1^ × mg^−1^ protein

### 3.8. Protein Carbonylation

The carbonyl group content was measured following the method described by Levine et al. [60]. Leaf samples weighing 400 mg were homogenized in 1.2 mL of cold 50 mM sodium phosphate buffer (pH 7.4), containing 1 mM of EDTA. The homogenate was then centrifuged at 6000× *g* for 10 min at 4 °C. In regard to the resulting supernatant, 200 μL of the supernatant was added to 800 μL of 10 mM 2,4-dinitrophenylhydrazine (DNPH) in 2.5 M HCl. The blank samples consisted of 1 mL of 2.5 M HCl.

After incubating for one hour at room temperature in the dark, 1 mL of 20% (*w*/*v*) TCA was added to the samples. The samples were then cooled on ice for 5 min and centrifuged at 10,000× *g* for 10 min at 4 °C. The resulting pellet was washed three times with a 1:1 ethanol/ethyl acetate mixture, vortexed, and centrifuged again at 10,000× *g* for 10 min at 4 °C.

The pellet was subsequently dissolved in 1 mL of 6 M guanidine hydrochloride in 20 mM potassium phosphate buffer (pH 2.3). The samples were centrifuged at 10,000× *g* for 10 min at 4 °C. The absorbance was measured at 375 nm using a UV–VIS Spectrophotometer Pharo 300 Spectroquant (Merck, Darmstadt, Germany). The carbonyl group content was calculated using a molar absorption coefficient for aliphatic hydrazones of 22,000 M^−1^ cm^−1^ and expressed in nM carbonyl per mg of protein (nM × mg^−1^ protein).

### 3.9. Total RNA Extraction

The isolation of the total RNA from barley shoots was performed using the GeneMATRIX Universal RNA Purification Kit (EURx), according to the manufacturer’s instructions. This procedure included an additional treatment with RNase-free DNase I. The RNA content was estimated spectrophotometrically using an Eppendorf BioSpectrometr^®^ basic, while its purity and integrity were verified by loading 1.5% (*w*/*v*) agarose gel and running horizontal electrophoresis in 1 × TBE buffer (89 mM Tris, 89 mM boric acid, 2 mM EDTA, pH 8.3).

For reverse transcription, 1 μg of the extracted total RNA samples was used in regard to the High-Capacity cDNA Reverse Transcription Kit (Thermo Scientific). The thermal cycler conditions utilized were as follows: 10 min at 25 °C, 120 min at 37 °C, 5 min at 85 °C, and finally 10 min at 4 °C.

### 3.10. Quantitative Reverse Transcription PCR (Q-RT PCR)

A quantitative reverse transcription PCR (q-RT PCR) was performed using LightCycler 8-Tube Strips (Roche Diagnostics) on the LightCycler^®^ 96 instrument (Roche Diagnostics). The reaction mixture was prepared following the standard protocol for the FastStart Essential DNA Green Master, also produced by Roche Diagnostics. The mixture included 1 μL of PCR-grade water, 0.5 μL of each primer (at a concentration of 0.1 mM), 5 μL of Master Mix, and 3 μL of a first-strand cDNA template, diluted at a ratio of 1:7.

The thermocycling conditions were set as follows: an initial step of 6 min at 95 °C, followed by 40 cycles of 10 s at 95 °C, 15 s at 58 °C, and 10 s at 72 °C. To verify the specificity of the amplification, melting curves were generated for each reaction, consisting of a 10 s hold at 95 °C, a 60 s hold at 65 °C, and a final 1 s hold at 95 °C.

Relative gene expression (RQ) was calculated using the formula 2^−ΔΔCT^ [61]. All relative expression levels were log_2_ transformed, and the gene encoding actin was utilized as an internal control. A list of all the primers used for the real-time PCR can be found in Table 1.

### 3.11. Statistical Analysis

Representative experimental data from three independent biological replicates are presented as the mean ± standard deviation (SD). Statistical significance was assessed using a two-way analysis of variance (ANOVA), followed by Tukey’s honest significant difference test (*p* < 0.05). Pearson’s correlation coefficients were calculated to determine the relationships between the observed traits (*p* < 0.05). All the statistical analyses were performed using GraphPad Prism 10 (GraphPad Software Inc., La Jolla, CA, USA).

## 4. Conclusions

Our research investigated the effects of Ba on the physiological responses of spring barley (*Hordeum vulgare* L.), with a particular emphasis on nitro-oxidative pathways. The findings demonstrated significant alterations in the expression of genes encoding antioxidant enzymes and increased hydrogen peroxide (H_2_O_2_) levels in response to Ba. Notably, exposure to lower concentrations of Ba, along with a shorter duration, resulted in elevated nitric oxide (NO) levels. In contrast, higher concentrations of Ba and extended exposure durations led to a reduction in NO levels, thereby influencing nitrogen metabolism. This study elucidates the risks associated with Ba exposure in cultivated species and highlights the need for further research to clarify its impact on plant physiology and potential implications for human health.

## Figures and Tables

**Table 1:** Sequence of primers used in the q-RT PCR analysis; “-” in source column means sequences projected by the authors.

Gene	Accession Number	Sequence	Source
*Actin*	AY145451.1	F: TGCCATGTACGTCGCTATTC R: GCTTCTCCTTGATGTCCCTTAC	-
*CAT*	U20778.1	F: TGCAGGAGTACTGGCGTCTTCGACTT R: AGATCCCGGGCACGAGGCCGGGGCC	[62]
*SOD*	AK252295	F: ATGGTGAAGGCTGTTGCTGTGC R: TCAGCCTTGAAGTCCGATGATCCC	[62]
*APX*	AJ006358	F: GGAGTTGTCGCCGTGGAGGTGTCCGGTG R: CAAGATCACCCTGGTCGCGCATAGTAGC	[62]
*NR*	X57844.1	F: CGACGAGATACTACCCATCAAC R: TCGATCTCTACCGACCAGAA	-
*NiR*	X57844.1	F: GCTGCCTCACCAAGAACA R: ATCTTTGGGCTCCGACAAC	-

## Data Availability

The data are contained within the article.

## Abbreviations

The following abbreviations are used in this manuscript:

APX	Ascorbate Peroxidase
CAT	Catalase
DAF-FM DA	4-Amino-5-Methylamino-2′,7′-Difluorofluorescein Diacetate
DNPH	2,4-Dinitrophenylhydrazine
EDTA	Ethylenediaminetetraacetic Acid
NiR	Nitrite Reductase
NR	Nitrate Reductase
PMSF	Phenylmethylsulfonyl Fluoride
PPFD	Photosynthetic Photon Flux Density
PVP	Polyvinylpyrrolidone
ROS	Reactive Oxygen Species
SOD	Superoxide Dismutase
TBA	2-Thiobarbituric Acid
TBARS	2-Thiobarbituric Acid Reactive Substances
TCA	Trichloroacetic Acid
TEMED	Tetramethylethylenediamine
